# RNA-Seq analysis and targeted mutagenesis for improved free fatty acid production in an engineered cyanobacterium

**DOI:** 10.1186/1754-6834-6-113

**Published:** 2013-08-06

**Authors:** Anne M Ruffing

**Affiliations:** 1 Sandia National Laboratories, Department of Bioenergy and Defense Technologies, MS 1413, P.O. Box 5800, 87185-1413, Albuquerque, NM, USA

**Keywords:** Free fatty acid biosynthesis, FFA biosynthesis, Cyanobacterial biofuels, Algal biofuels, Cyanobacteria, Free fatty acid, RNA-seq, FFA toxicity

## Abstract

**Background:**

High-energy-density biofuels are typically derived from the fatty acid pathway, thus establishing free fatty acids (FFAs) as important fuel precursors. FFA production using photosynthetic microorganisms like cyanobacteria allows for direct conversion of carbon dioxide into fuel precursors. Recent studies investigating cyanobacterial FFA production have demonstrated the potential of this process, yet FFA production was also shown to have negative physiological effects on the cyanobacterial host, ultimately limiting high yields of FFAs.

**Results:**

Cyanobacterial FFA production was shown to generate reactive oxygen species (ROS) and lead to increased cell membrane permeability. To identify genetic targets that may mitigate these toxic effects, RNA-seq analysis was used to investigate the host response of *Synechococcus elongatus* PCC 7942. Stress response, nitrogen metabolism, photosynthesis, and protein folding genes were up-regulated during FFA production while genes involved in carbon and hydrogen metabolisms were down-regulated. Select genes were targeted for mutagenesis to confirm their role in mitigating FFA toxicity. Gene knockout of two porins and the overexpression of ROS-degrading proteins and hypothetical proteins reduced the toxic effects of FFA production, allowing for improved growth, physiology, and FFA yields. Comparative transcriptomics, analyzing gene expression changes associated with FFA production and other stress conditions, identified additional key genes involved in cyanobacterial stress response.

**Conclusions:**

A total of 15 gene targets were identified to reduce the toxic effects of FFA production. While single-gene targeted mutagenesis led to minor increases in FFA production, the combination of these targeted mutations may yield additional improvement, advancing the development of high-energy-density fuels derived from cyanobacteria.

## Background

Free fatty acids (FFAs) have recently garnered attention as potential precursors for renewable fuel production. FFAs are ideal targets for biofuel production: the pathway for FFA biosynthesis is already present in microorganisms for membrane production [1]; FFAs are passively excreted outside the cell for easy extraction without cell harvesting [2,3]; and FFAs can be readily converted into high-energy-density fuels [4]. Recent efforts in microbial FFA production have focused on the conversion of sugar feedstocks into FFAs using heterotrophic microorganisms such as *Escherichia coli*[5] and on the conversion of carbon dioxide into FFAs using photosynthetic microorganisms such as the cyanobacterial species, *Synechocystis* sp. PCC 6803 and *Synechococcus elongatus* PCC 7942 [6,7]. While these efforts have been successful in producing FFAs, overall productivities are limited by the toxic effects of FFA synthesis.

Exogenous FFAs are toxic to many organisms, including Gram-negative and Gram-positive bacteria, microalgae, fungi, protozoans, and multi-cellular organisms [8]. In fact, FFA production has been documented as an antimicrobial defense mechanism in multicellular organisms. The primary targets of FFA toxicity are cellular membranes, whereby FFAs disrupt the electron transport chain and oxidative phosphorylation, damaging cellular energy production. FFAs are also believed to inhibit enzyme activity, impair nutrient uptake, and alter cell membrane permeability [8]. In microalgae, cell lysis was observed following FFA addition along with dissociation of the phycobilisomes from the thylakoid membrane [9]. Evidence of these toxic effects was also found in FFA-producing cyanobacteria for biofuel production. FFA-producing strains of *S. elongatus* PCC 7942 had reduced photosynthetic yields, suggestive of electron transport disruption, and the phycobiliproteins of these strains were aggregated at the cell poles rather than evenly dispersed throughout the thylakoid membranes, indicating dissociation of the phycobiliosomes [7]. FFA-producing strains of *Synechocystis* sp. PCC 6803 also had increased membrane permeability [6]. Unsaturated fatty acids (UFAs) have generally been found to be more toxic than their saturated counterparts [8]; this was confirmed in *S. elongatus* PCC 7942 by the exogenous addition of both saturated fatty acid (SFA) and UFA [7]. Only UFA addition yielded negative physiological effects, while no effect was observed with SFA addition. The physiological effects associated with exogenous UFA addition, however, were found to differ from the effects observed with FFA biosynthesis, suggesting that the mechanism of toxicity may also differ.

While the mechanism(s) involved in FFA toxicity remain unproven, two possible mechanisms have been proposed based on the available evidence: (1) UFAs may react with reactive oxygen species (ROS) to generate toxic products such as hydroperoxides, free radical species, aldehydes, and oxylipins, and (2) the amphipathic structure of FFAs may allow them to intercalate into both the cell and thylakoid membranes [8]. The generation of toxic products will cause widespread cellular damage due to the chemical reactivity of the generated products, while the intercalation of FFAs in membranes will inhibit membrane proteins and destabilize membrane structure. The two proposed mechanism may even act in concert, with the intercalation of FFAs in the thylakoid membrane disrupting photosynthetic electron transport, leading to excess light collection and subsequent ROS generation. These potentially complex mechanisms of FFA toxicity make this issue difficult to resolve, yet this obstacle must be overcome to enable FFA-based biofuel production.

In order to surmount the physiological effects of FFA production, the underlying mechanisms of FFA toxicity must be investigated along with the specific host response. This study explored the two proposed mechanisms of FFA toxicity through analysis of ROS generation and cell membrane permeability. The global transcriptional response of the cyanobacterial host, *S. elongatus* PCC 7942, to FFA production was quantified using RNA-seq, and from this analysis, differentially expressed genes were identified for either gene knockout or gene overexpression. The physiological responses and FFA productivities of these targeted mutants were assessed to determine the gene’s role in cellular FFA toxicity response. Additionally, the transcriptional response to FFA production was compared to other transcriptomic studies of cyanobacterial stress response to identify the key genes involved in general cyanobacterial stress response. From the analyses presented in this study, genetic targets were determined for improving cyanobacterial physiology during FFA production.

## Results and discussion

### Physiological effects of FFA production are linked to cell stress and membrane permeability

Previously, *S. elongatus* PCC 7942 was engineered for FFA production by gene knockout of acyl-ACP synthetase (SE01 = *S. elongatus* PCC 7942 Δ*aas*) and introduction of a truncated *E. coli* thioesterase (SE02 = *S. elongatus* PCC 7942 Δ*aas ‘tesA*) [7]. Knockout of *aas* was necessary to prevent recycling of FFAs through the β-oxidation pathway, while expression of the thioesterase released FFAs from acyl-ACP. Synthesis and excretion of FFAs resulted in detrimental physiological effects, ultimately limiting FFA biosynthesis. The two potential mechanisms of FFA toxicity, described previously, were investigated to determine the underlying cause of the observed physiological effects in the FFA-producing strains of *S. elongatus* PCC 7942, SE01 and SE02.

**Figure 1:**
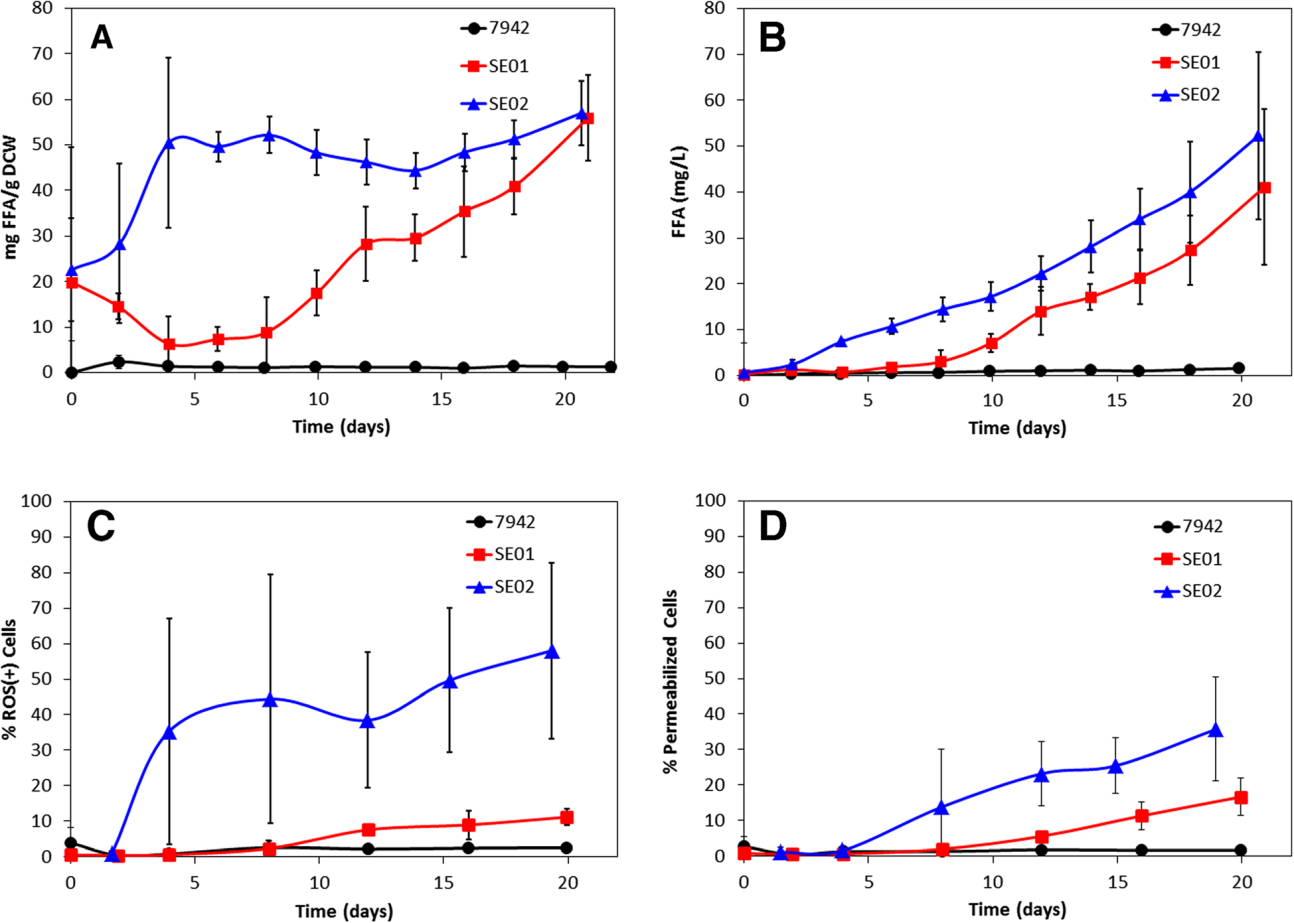
**FFA production and physiological measurements for wild-type (7942) and FFA-producing strains (SE01 and SE02): (A) mg of FFA per gram of dry cell weight; (B) FFA concentration in mg/L; (C) percentage of cells staining positive for ROS; (D) percentage of cells with permeable membranes.** Data are averages of 3 biological replicates with error bars indicating the standard deviation of these measurements.

ROS levels were measured in both the wild type and FFA-producing strains to determine whether these species were present for UFA degradation. As illustrated in Figure 1C, ROS generation in the wild type (7942) was negligible (< 2.56 ± 0.65%), yet both FFA-producing strains, SE01 and SE02, had intracellular ROS accumulation (up to 11.1 ± 2.3% and 58.1 ± 24%, respectively). The highest ROS levels were measured in SE02, with nearly 60% of the cell population staining positive for ROS at day 20. In general, the measurement of ROS-positive cells trended with FFA production (7942 < SE01 < SE02), and there was a statistically significant correlation between the amount of FFA on a per cell weight basis and the percentage of ROS-positive cells (R^2^ = 0.46, *p* = 0.0367) (Figure 1 A and C). However, the values of FFA concentration (mg/L) and the percentage of ROS-positive cells did not correlate (R^2^ = 0.13, *p* = 0.721) (Figure 1 B and C), suggesting that ROS generation is due to intracellular FFAs rather than extracellular. The accumulation of ROS during FFA production may indicate a potential for UFA degradation into toxic products, as described in the first proposed mechanism; however, ROS accumulation is also a hallmark of cellular stress [10]. Thus, we are presented with the ‘chicken or the egg’ dilemma, do the UFAs react with ROS to produce compounds that are toxic to the host cell, or do the FFAs themselves cause cellular stress which leads to ROS generation? The fact that there is no ROS accumulation in the wild type suggests that the latter hypothesis is correct.

The amphipathic character of FFAs may allow them to intercalate into cellular membranes, altering membrane fluidity, membrane integrity, and the activity of membrane-bound proteins, such as those involved in photosynthesis. To investigate this proposed mechanism of FFA toxicity, a membrane impermeable nucleic acid stain, SYTOX, was used to interrogate membrane permeability in the wild type and FFA-producing strains. Similar to ROS accumulation, cell membrane permeability trended with FFA production (7942 < SE01 < SE02) (Figure 1D). Additionally, the percentage of membrane-permeable cells had statistically significant correlation with the amount of FFA per gDCW (R^2^ = 0.48, *p* = 2.10 x 10^-4^) (Figure 1B and D), suggesting that intracellular FFA production also influences membrane permeability.

Based on this preliminary assessment, both proposed mechanisms of FFA toxicity are possible and may be contributing factors of the observed toxicity. So the question remains, how can these toxic effects be prevented or mitigated? Microorganisms have evolved a multitude of mechanisms for coping with stressful conditions, including both general and stress-specific response mechanisms [11]. Such natural mechanisms may be activated in *S. elongatus* PCC 7942 with FFA production. Furthermore, some strains of microalgae are known to excrete a polyunsaturated fatty acid (PUFA) as a defense mechanism against grazers [12]. Along with this defense mechanism, microalgae have likely evolved mechanisms to protect against PUFA toxicity. Therefore, RNA-seq analysis was applied to investigate natural mechanisms of FFA toxicity defense in *S. elongatus* PCC 7942; amplification of these natural responses may allow for increased FFA production.

### RNA-seq analysis of FFA production

To investigate the transcriptional response of *S. elongatus* PCC 7942 to FFA production, both the wild type (7942) and FFA-producing (SE01 and SE02) cultures were sampled at 100 h and 240 h for RNA-seq analysis. Genes differentially expressed during high FFA production were identified through six different comparisons: A) SE02:SE01 at 100 h, B) SE02:7942 at 100 h, C) SE01:7942 at 240 h, D) SE02:7942 at 240 h, E) 240 h:100 h for SE01, and F) 240 h:100 h for SE02. It should be noted that comparisons A and B are only valid for increased extracellular FFA production on a cell weight basis (mg/gDCW), while comparison F is only valid for enhanced extracellular FFA concentration (mg/L). All other comparisons (C, D, and E) apply to both measurements of FFA production (Figure 1 A and B). Differentially expressed genes were defined as having fold changes greater than 2; these genes are listed in Tables S1 and S2 in Additional file 1 along with their expression fold changes and associated *p*-values. Combining results from the six comparisons, 228 genes were up-regulated and 223 genes were down-regulated with increased FFA production (Table 1). The largest functional gene category for both up- and down-regulated genes was hypothetical proteins, comprising approximately 45% of the total quantity of differentially expressed genes.

**Table 1:** **Number of differentially expressed genes in *S. elongatus *PCC 7942 during FFA production based on predicted gene function**

**Gene Function**	**Up-regulated**		**Down-regulated**		**Total**	
	**# Genes**	**%**	**# Genes**	**%**	**# Genes**	**%**
Stress Response	11	4.8	11	4.9	22	4.9
Photosynthesis	10	4.4	16	7.2	26	5.8
Electron Transport	10	4.4	8	3.6	18	4.0
Carbon Metabolism	16	7.0	13	5.8	29	6.4
Nitrogen Metabolism	7	3.1	2	0.9	9	2.0
Hydrogen Metabolism	5	2.2	7	3.1	12	2.7
Protein Biosynthesis & Processing	8	3.5	7	3.1	15	3.3
Nucleotide Biosynthesis & Metabolism	10	4.4	5	2.2	15	3.3
Transporters	14	6.1	10	4.5	24	5.3
Regulatory Proteins	7	3.1	19	8.5	26	5.8
c-di-GMP Associated Proteins	1	0.4	7	3.1	8	1.8
Cell Wall Biosynthesis	7	3.1	3	1.3	10	2.2
Other	22	9.6	14	6.3	36	8.0
Hypothetical Proteins	100	43.9	101	45.3	201	44.6
**Totals**	**228**	**100.0**	**223**	**100.0**	**451**	**100.0**

#### Up-regulated transcript expression

Genes showing increased expression during elevated FFA production were predominantly associated with known cellular stress responses. General stress response genes such as high light-inducible proteins (Synpcc7942_1997 and *hliC*) and heat shock proteins (*hsp20* and *hsp90*) were significantly up-regulated with FFA production. As described in the previous section, FFA production was accompanied by elevated ROS levels. To minimize ROS-induced cellular damage, oxidative stress response genes were elevated during FFA production, including ROS-degrading enzymes such as superoxide dismutase (Synpcc7942_0801) and glutathione peroxidases (Synpcc7942_1214 and Synpcc7942_0437). High light-inducible proteins have also been shown to enhance the ability of cyanobacteria to cope with oxidative stress response under high light [13]. The production of carotenoids to absorb excess light energy and prevent photooxidative damage is yet another stress response mechanism in cyanobacteria [14]. Phytoene dehydrogenase, a key enzyme in carotenoid biosynthesis, was also up-regulated with FFA production. Enhanced transcription of these stress response mechanisms may contribute to the survival of SE01 and SE02 during FFA production.

Unexpectedly, many genes associated with nitrogen metabolism were up-regulated (Table 1). Three genes associated with nitrate transport were elevated during FFA production along with a nitrate reductase and ferredoxin-nitrite reductase, constituting the nitrogen acquisition pathway. Additionally, adenosine deaminase, an enzyme involved in intracellular nitrogen scavenging, showed enhanced expression, suggesting that FFA-producing cells had activated a nitrogen limitation response. Biochemical data also supports this perceived nitrogen limitation response, with selective degradation of the nitrogen-containing chlorophyll-*a* pigment [7,15]. With sufficient levels of nitrate in the media, the signal for activation of this nitrogen-limited response with FFA production remains unknown; however, nutrient acquisition and storage may simply be a general, conserved stress response in *S. elongatus* PCC 7942.

Increased FFA production also affected protein production, with elevated transcript levels of the *groEL-groES* chaperone system (Additional file 1: Table S1). The GroEL-GroES system was also up-regulated under high light stress in other cyanobacterial species [16], suggesting that this too may be a conserved stress response. Moreover, GroEL and other molecular chaperonins are required for the insertion and stabilization of membrane proteins and ribulose-1,5-bisphosphate carboxylase/oxygenase (RuBisCO) assembly [17]. In a previous study [7], we observed reduced growth rates and photosynthetic yields concomitant with FFA production, which may result from impaired photosynthesis and carbon fixation, inactivation of electron transport in the thylakoid membrane, or reduced binding affinity of phycobilisomes (PBSs) to the thylakoid membranes. As cyanobacterial photosynthesis, electron transport, and light harvesting all involve integral membrane or membrane-associated proteins, the *groEL* and *groES* transcripts may be elevated in response to the low activity of these membrane processes.

Transcripts associated with photosynthesis were significantly up-regulated during FFA production. The RNA polymerase sigma factor, *sigD* (Synpcc7942_0672), had elevated expression; SigD is a light-inducible sigma factor which activates transcription of *psbA*, the D1 protein of photosystem II (PSII) [18]. In agreement with the elevated *sigD* transcript levels, a PSII D1 protein (Synpcc7942_0893) showed enhanced expression. Many other PSII proteins were up-regulated, including several PSII D2 proteins (Synpcc7942_1637 and Synpcc7942_0655) and the PSII reaction center protein N (*psbN*). In 3 out of the 6 comparisons, transcript levels of the PBS degradation protein, *nblA*, were significantly increased by an average of 4.37-fold. Phycobiliprotein levels, however, did not change significantly in the FFA-producing cultures [7].

A potential solution to the issue of FFA toxicity is to overexpress FFA export proteins, effectively removing the toxic product from the intracellular space. Several efflux pumps in *E. coli*, particularly TolC-AcrAB, have been identified as potential FFA exporters, although increased expression of these genes did not improve FFA production in the *E. coli* host [19]. While there is no significant *tolC* homolog in *S. elongatus* PCC 7942, a hydrophobe/amphiphile efflux protein (Synpcc7942_2369) was found to be homologous to several *E. coli* efflux proteins including *acrA, acrB, acrD, acrF, mdtB,* and *mdtF.* However, gene expression of Synpcc7942_2369 was not significantly changed during FFA production. Several other transport proteins and porin proteins were found to be up-regulated during FFA production (Additional file 1: Table S1). While these proteins may contribute to FFA export, there was no consensus among the 6 comparisons of high v. low FFA production, with potential FFA export proteins showing a conserved expression enhancement in only 2 of the 6 comparisons.

#### Down-regulated transcript expression

In addition to the up-regulation of genes involved in stress response and photosynthesis, genes within these functional categories were also significantly down-regulated (Tables 1 and Additional file 1: Table S2). Across the 6 comparisons, however, there was little consensus among the down-regulated stress response genes, implying that repression of stress response proteins is not a conserved response to FFA production. On the other hand, there was some consensus to support the reduced expression of genes involved in photosynthesis. In particular, the light-independent protochlorophyllide reductase subunit B was down-regulated in 4 out of the 6 comparisons. This protein is involved in light-independent chlorophyll biosynthesis [20], and decreased expression of this gene may contribute to the measured decrease in chlorophyll-*a* content for the FFA-producing cultures [7]. Other photosynthetic genes with reduced expression include components of photosystem I (PSI), specifically PSI reaction center subunits X and XII. The down-regulation of PSI components along with up-regulation of PSII components suggests an imbalance of electron distribution between PSI and PSII. Previous hyperspectral imaging of FFA-producing cells indicated that the PBSs were no longer attached to the thylakoid membranes. As PBSs are the light-harvesting pigments for PSII [21], this detachment would reduce electron flow through PSII, possibly leading to the measured transcriptional changes to restore the PSI:PSII balance. Conserved down-regulation of cytochrome associated proteins, cytochrome aa3 controlling protein and cytochrome c oxidase subunit II, may represent another cellular mechanism to restore the electron flow balance. PBS detachment would also explain the up-regulation of *nblA* to degrade the non-functional PBSs. Transcriptional repression of genes involved in chlorophyll biosynthesis and electron flow distribution support biochemical measurements of reduced chlorophyll-*a* pigment concentration and decreased photosynthetic yield measurements [7].

Another conserved response of transcriptional repression is associated with carbon metabolism. Specifically, phosphoenolpyruvate (PEP) synthase was significantly down-regulated an average of 3.87-fold in 5 of the 6 comparisons. PEP synthase catalyzes an energy-consuming reaction, and reduced expression of this enzyme may serve as a stress-induced mechanism for energy conservation. Repression of PEP synthase also increases carbon flux through the glycolysis pathway, which feeds into the fatty acid biosynthesis pathway. Thus, the lower level of fatty acids available for membrane biosynthesis, due to fatty acid cleavage by the recombinant thioesterase, may cause the cell to increase carbon flux through the fatty acid biosynthesis pathway via PEP synthase repression. A sodium-dependent bicarbonate transporter showed an average 9-fold decrease in transcript level for 3 comparisons, suggesting a decrease in photosynthetic carbon fixation, and subsequently, a lower overall carbon flux may accompany FFA production. Lastly, the long-chain-fatty-acid CoA ligase or acyl ACP synthetase (*aas*) was also down-regulated in comparisons B, C, and D. As you may recall, this gene was targeted for gene knockout in both SE01 and SE02. Therefore, the reduced transcription of *aas* in only comparisons of the wild type with the engineered strains (i.e. B, C, and D) validates the data processing protocol developed for the RNA-seq analysis. Repression of carbon metabolism in FFA-producing cultures, as suggested by RNA-seq analysis, may serve as yet another indicator of severe cellular stress.

Genes associated with hydrogen metabolism had reduced transcript levels during FFA production. The genes encoding the hydrogenase accessory proteins, *hypE* and *hypD*, were repressed in multiple comparisons (Additional file 1: Table S2). Transcriptional changes in hydrogen metabolism were not expected due to the fact that hydrogen biosynthesis is predominantly active only under anaerobic conditions as a result of the inhibitory effect of oxygen [22]. The hydrogenase in *S. elongatus* PCC 7942 (*hoxEF* and *hoxUYH*), however, is a bidirectional hydrogenase with undetermined physiological function. Proposed functions for this hydrogenase includes the delivery of electrons into respiratory complex I and the regulation of photosynthetic electron flow [23]. Repression of hydrogenase-associated genes may therefore result from the reduced electron flow in PSII due to FFA production, as evidenced by low photosynthetic yields [7].

Many regulatory proteins were down-regulated across multiple comparisons as well as genes associated with the global signaling molecule, cyclic-di-guanosylmonophosphate (c-di-GMP). Cellular c-di-GMP levels have been linked to the regulation of a variety of cellular processes, including cellular motility, virulence, heavy metal resistance, phage resistance, cell-cell communication, photosynthesis, exopolysaccharide production, and biofilm formation [24]. The reduced expression of these c-di-GMP proteins was almost exclusively associated with comparisons A and B, suggesting that c-di-GMP plays a unique role in early FFA production in SE02. Further exploration of the role of c-di-GMP in FFA production may provide insight into the increased toxicity observed in the SE02 strain.

### Genetic targets for improved FFA production

In an effort to identify potential targets for reducing the toxic effects of FFAs and improving FFA production, differentially expressed genes were targeted for either gene knockout or gene overexpression in SE02. SE02 was selected as the host strain for these targeted mutations because *aas* deletion is required to prevent FFA recycling and SE02 had increased physiological effects during FFA production. The gene targets included hypothetical proteins, ROS-degrading proteins, and possible FFA exporters (Table 2). Nine hypothetical proteins were targeted which showed differential gene expression in 4 of 5 comparisons (excluding comparison F). The three hypothetical proteins showing a significant increase in gene expression were targeted for gene knockout while the six down-regulated hypothetical proteins were overexpressed. These hypothetical protein mutants were expected to have decreased FFA production if the gene target is involved in either FFA production or FFA toxicity mitigation. Four ROS-degrading proteins, 2 glutathione peroxidases, a superoxide dismutase, and catalase, were also targeted for overexpression. As elevated ROS production was found to correlate with FFA production (Figure 1), these mutants were expected to show improved FFA tolerance. Lastly, four potential FFA exporters, 2 porins and 2 transport proteins, were targeted for gene knockout. If these export proteins participate in FFA export, the associated knockout mutants should show reduced extracellular FFA concentrations and possibly an increased toxic response to FFA production.

**Table 2:** **Differentially expressed genes in *S. elongatus *PCC 7942 during FFA production selected for targeted mutagenesis**

**Locus**	**Product**	**Average FC**	**Targeted Mutagenesis**
*Hypothetical Proteins*			
Synpcc7942_0444	hypothetical protein	3.27	Knockout
Synpcc7942_1561	hypothetical protein	2.67	Knockout
Synpcc7942_1023	hypothetical protein	2.15	Knockout
Synpcc7942_1476	hypothetical protein	−4.59	Overexpression
Synpcc7942_B2645	hypothetical protein	−7.35	Overexpression
Synpcc7942_1655	hypothetical protein	−2.98	Overexpression
Synpcc7942_0900	hypothetical protein	−2.92	Overexpression
Synpcc7942_B2632	hypothetical protein	−2.68	Overexpression
Synpcc7942_0122	hypothetical protein	−2.53	Overexpression
Synpcc7942_1845	hypothetical protein	−2.28	Overexpression
*ROS-Degrading Proteins*			
Synpcc7942_1214	glutathione peroxidase	2.63	Overexpression
Synpcc7942_0801	superoxide dismutase	2.56	Overexpression
Synpcc7942_0437	glutathione peroxidase	2.54	Overexpression
Synpcc7942_1656	catalase/peroxidase HPI	−2.38	Overexpression
*Potential FFA Exporters*			
Synpcc7942_2175	transport system substrate-binding protein	2.99	Knockout
Synpcc7942_1224	ABC-transporter membrane fusion protein	2.74	Knockout
Synpcc7942_1464	porin	2.33	Knockout
Synpcc7942_1607	porin; major outer membrane protein	2.16	Knockout

Average fold change (FC) values are calculated by averaging the fold change associated with differentially expressed genes under the 6 different comparisons of high v. low FFA production.

Out of the 17 targeted mutants, 15 were successfully constructed. The knockout mutants for the hypothetical proteins Synpcc7942_1561 and Synpcc7942_1023 could not be obtained despite repeated transformation attempts, indicating that these genes may be essential for cell growth. The 15 constructed mutants were screened in shake-flask experiments to determine the effect of the targeted mutation on FFA production and cell physiology. For each mutant, 2 transformants were analyzed to account for biological variation. Additionally, SE02a was constructed as a control by integrating the empty vector, pSA, into SE02 at neutral integration site II (NSII).

**Figure 2:**
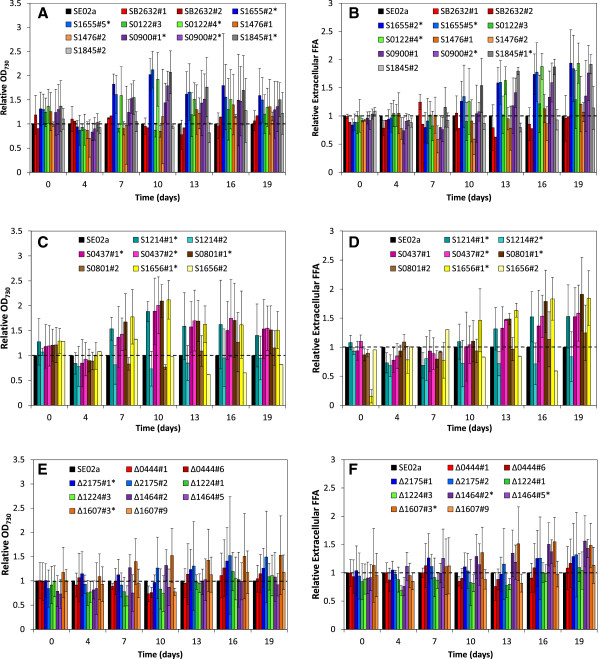
**Cell concentration and extracellular FFA concentration of targeted mutants relative to the control strain, SE02a. (A)** relative cell concentration of mutants overexpressing hypothetical proteins; **(B)** relative extracellular FFA concentration of mutants overexpressing hypothetical proteins; **(C)** relative cell concentration of mutants overexpressing ROS-degrading proteins; **(D)** relative extracellular FFA concentration of mutants overexpressing ROS-degrading proteins; **(E)** relative cell concentration of knockout mutants; **(F)** relative extracellular FFA concentration of knockout mutants. Knockout mutants include one hypothetical protein mutant (Δ0444) and 4 putative transport proteins (Δ2175, Δ1224, Δ1464, and Δ1607). Data are averages of 3 biological replicates with error bars indicating the standard deviation of these measurements, with a few exceptions: Due to strain instabilities, mutants SB2632#1 and S1476#1 have only 2 biological replicates, and mutants SB2632#2 and S1656#2 have only 1 biological replicate. The dashed line at y = 1 indicates no change relative to the control, SE02a. An asterisk (*) following the mutant strain name in the legend indicates that difference between the control (SE02a) and mutant strain was determined to be statistically significant by ANOVA analysis (see Table S7 in Additional file 3 for calculated *p*-values).

Significant changes in relative cell concentration occurred most frequently for days 7, 10, and 13, following induction of the recombinant proteins (Figure 2 A, C, and E). The hypothetical protein overexpression mutants S1655#2 and #5, S0122#4, S0900#1 and #2, and S1845#1, along with the ROS-degrading protein overexpression mutants S1656#1, S0801#1, S0437#1 and #2, and S1214#1, had increased cell concentrations relative to SE02a after recombinant protein induction. Improved growth of the hypothetical protein overexpression mutants was unexpected, as these genes were repressed during FFA production. On the other hand, overexpression of ROS-degrading genes was expected to improve cellular stress resistance and overall fitness; the enhanced relative cell concentrations of the ROS-degrading mutants provide evidence to support this theory. The knockout mutants Δ2175#1 and Δ1607#3 also had statistically significant increases in relative cell concentration. As putative FFA exporters, these knockout mutants were anticipated to have higher FFA toxicity and therefore lower relative cell concentrations. While relative cell concentrations showed significant changes after induction, these differences were reduced over time, so that at the end of cultivation (day 19), only a few mutants maintained a significant deviation in relative cell concentration (Figure 2 A, C, E).

Changes in relative extracellular FFA concentration were found to correlate with the changes in relative cell concentration for the hypothetical protein and ROS-degrading protein overexpression mutants; the mutants showing increased relative cell concentrations also had increased relative extracellular FFA concentrations (Figure 2 B and D). This relationship between relative cell concentration and increased extracellular FFA concentration would be expected if the rate of FFA production was similar on a per cell basis. Unexpectedly, however, there appears to be a 6 day delay between increased cell concentration (days 7, 10, and 13) and enhanced FFA concentration (days 13, 16, and 19). In addition, the correlation between relative cell concentration and relative extracellular FFA concentration does not always hold true. The porin knockout mutants, Δ1464#2 and #5, showed increased relative extracellular FFA concentration (Figure 2F) without having an improvement in relative cell concentration (Figure 2E). The other porin mutant Δ1607#3 also had improved FFA production. Enhanced extracellular FFA concentration in the Δ1464 and Δ1607 transformants suggests that these porins may actually contribute to FFA uptake rather than export. In general, only small improvements in relative extracellular FFA concentrations were observed for the 15 mutants, yet by targeting these multiple genes in parallel, further gains in FFA productivity may be realized.

**Figure 3:**
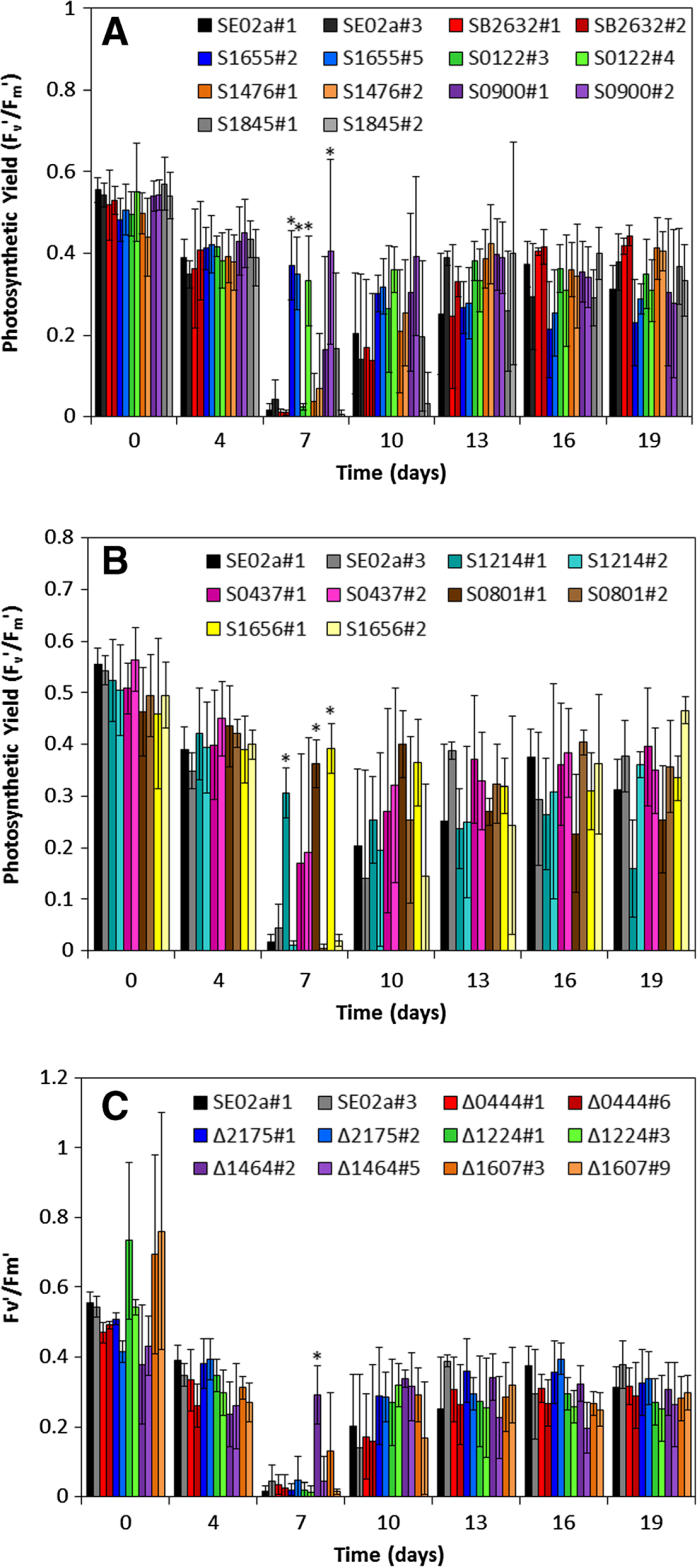
**Photosynthetic yield measurements for the targeted mutants: (A) hypothetical protein overexpression mutants; (B) ROS-degrading protein overexpression mutants; (C) knockout mutants, including include one hypothetical protein mutant (Δ0444) and 4 putative transport proteins (Δ2175, Δ1224, Δ1464, and Δ1607).** Data are averages of 3 biological replicates with error bars indicating the standard deviation of these measurements. An asterisk (*) above the data for day 7 indicates that difference between the control (SE02a) and mutant strain was determined to be statistically significant by ANOVA analysis (see Table S7 in Additional data file 3 for calculated *p*-values).

Photosynthetic yield measurements were analyzed to serve as an estimate of overall cell physiology. For the control strain SE02a, photosynthetic yield declined severely after induction at day 4, with photosynthetic yield measurements approaching zero at day 7 (Figure 3). Hypothetical protein overexpression mutants (S1655#2 and #5, S0122#4, and S0900#2), ROS-degrading protein overexpression mutants (S1214#1, S0801#1, and S1656#1) and a porin knockout mutant (Δ1464#2) all showed significantly elevated photosynthetic yields at day 7, ranging from 0.3 to 0.4 (Figure 3). Not surprisingly, these mutants also had increased relative cell concentrations and relative extracellular FFA concentrations, with the exception of Δ1464#2, which had increased relative extracellular FFA concentrations but no change in relative cell concentration (Figure 2). The general positive correlation between the three measured parameters of cell concentration, extracellular FFA concentration, and photosynthetic yield suggests that the corresponding genetic targets are involved in cellular defense mechanisms for FFA toxicity. While the ROS-degrading proteins are involved in well-known stress response mechanisms [10], the identification of 3 hypothetical proteins (Synpcc7942_1655, Synpcc7942_0122, and Synpcc7942_0900) and 2 porin proteins (Synpcc7942_1464 and Synpcc7942_1607) that counteract the toxic effects of FFA production provides new targets for improving cyanobacterial FFA production.

### Comparative transcriptomics identifies cyanobacterial stress response genes

In an attempt to determine which genes are involved in general cyanobacterial stress responses, the differentially expressed genes for FFA production identified in this study were compared to differentially expressed genes from previous studies investigating cyanobacterial stress responses. Specifically*, Synechococcus* sp. PCC 7002 transcriptome studies of temperature, oxidative, salt, and nutrient limitation (CO_2_, nitrogen, sulfur, phosphorous, and iron) stresses [25,26] were evaluated along with *Synechocystis* sp. PCC 6803 studies which analyzed transcriptional responses to temperature, oxidative, salt, osmotic, and ethanol stresses [27-34]. Homologous genes between *S. elongatus* PCC 7942, *Synechococcus* sp. PCC 7002, and *Synechocystis* sp. PCC 6803 were identified, as described in the Materials and Methods section, to facilitate the comparison. Many differentially expressed genes associated with FFA toxicity in *S. elongatus* PCC 7942 corresponded to homologs of differentially expressed genes in these previous stress studies (Additional file 2: Tables S3 and S4). Overall, the transcriptional response to FFA production in *S. elongatus* PCC 7942 was most similar to the heat stress response in *Synechococcus* sp. PCC 7002, with nearly 26% of the differentially transcribed genes associated with FFA toxicity having differentially expressed homologs in the heat stress study. Among the functional categories for differentially expressed genes, up-regulation of stress response and nitrogen metabolism genes was highly conserved among stress conditions, with 80% and 83% of stress response and nitrogen metabolism genes up-regulated during FFA production having at least one matching up-regulated stress homolog (Additional file 2: Table S3). Interestingly, the transcriptional response to nitrogen limitation in *Synechococcus* sp. PCC 7002 did not show any significant changes in genes associated with nitrogen metabolism [26], suggesting that the activation of nitrogen metabolism may be post-translational. The down-regulation of carbon and hydrogen metabolic genes was conserved among the cyanobacterial stress responses, with 80% and 100% of carbon and hydrogen metabolism genes repressed during FFA production having at least one corresponding homolog among the other stress conditions (Additional file 2: Table S4). This provides further evidence that *S. elongatus* PCC 7942 is highly stressed during FFA production, and it also suggests that conserved stress response mechanisms exist among cyanobacteria.

Genetic targets for improving FFA production in *S. elongatus* PCC 7942 include both general stress response genes as well as response mechanisms that may be specific for FFA-induced stress. Stress response genes that were highly conserved across the cyanobacterial transcriptomic studies also had high fold change values. In particular, two genes were differentially regulated in 13 of the compared stress conditions: the high light inducible protein (Synpcc7942_1997, average +11.47 fold change) and a hypothetical protein (Synpcc7942_0551, average −4.52 fold change). Another 4 genes were up-regulated in 11 or 12 of the stress conditions: the heat shock protein Hsp20 (Synpcc7942_2401, average +102.59 fold change), the NAD(P)H-quinone oxidoreductase subunit 4 (Synpcc7942_1439, average +10.54 fold change), RNA polymerase sigma factor SigD (Synpcc7942_0672, average +4.22 fold change), and Beta-Ig-H3/fasciclin (Synpcc7942_1606, average +12.44 fold change). These 6 genes are candidate targets for boosting the native stress response mechanisms of *S. elongatus* PCC 7942. The hypothetical protein and porin genes identified from targeted mutagenesis in the previous section were also investigated in this comparative transcriptomics analysis. Out of the 3 hypothetical protein targets (Synpcc7942_1655, Synpcc7942_0122, and Synpcc7942_0900), Synpcc7942_1655 was the only gene without homologs in *Synechococcus* sp. PCC 7002 and *Synechocystis* sp. PCC 6803. Both Synpcc7942_0122 and Synpcc7942_0900 had homologs in the other cyanobacterial species that were differentially expressed under other stress conditions, with down-regulation in 8 and 5 of the other stress conditions, respectively. Therefore, Synpcc7942_1655 may be a unique stress response gene in *S. elongatus* PCC 7942 or perhaps a response specific to FFA production, while Synpcc7942_0122 and Synpcc7942_0900 are likely involved in general stress response. The two porin targets, Synpcc7942_1464 and Synpcc7942_1607, were also differential expressed in the other cyanobacterial strains, showing up-regulation in 8 of the other stress conditions. The 9 genes identified through targeted mutagenesis and the 6 additional stress genes identified through this comparative transcriptomics analysis will be candidate targets for future improvement in cyanobacterial FFA production and general stress response.

## Conclusions

This study investigated two proposed mechanisms of FFA toxicity in cyanobacteria: UFA reactivity with ROS to produce toxic compounds and the intercalation of FFAs in cellular membranes. Elevated ROS levels in FFA-producing strains of *S. elongatus* PCC 7942 confirmed the presence of intracellular ROS, and based on the known reactive properties of UFAs and ROS [35], UFA degradation is anticipated. However, the amount of UFA degradation is unknown as well as the degree to which the degradation products cause cellular damage. In addition, the experimental results suggest that ROS generation is itself a result of FFA production, as the wild type does not have elevated ROS levels. This indicates that another mechanism of FFA toxicity causes cellular stress, leading to ROS generation. The FFA-producing strains also have increased cell membrane permeability, providing evidence to support the mechanism of FFA intercalation. In agreement with this, previous biochemical and imaging experiments showed reduced photosynthetic yields and dissociation of PBSs from the thylakoid membranes [7]. While further exploration of these mechanisms is necessary, these preliminary results point to FFA intercalation as a primary mechanism of intracellular FFA toxicity.

Through examining the transcriptional response of *S. elongatus* PCC 7942 to FFA production, natural mechanisms for alleviating FFA toxicity were identified. These mechanisms included the activation of genes contributing to general stress response, nitrogen metabolism, PSII photosynthesis, and protein folding as well as repression of genes involved in PSI photosynthesis and carbon and hydrogen metabolisms. Many of these cellular responses were expected, as they agree with the ‘core transcriptional response’ (CTR) genes determined for stress response in *Synechocystis* sp. PCC 6803 [36]. The differential expression of genes affecting electron transport, including PSI and PSII genes along with hydrogenase associated genes, provides additional evidence supporting the mechanism of FFA toxicity via membrane intercalation. The processes and genes identified in this RNA-seq analysis are potential targets for improving the physiology of *S. elongatus* PCC 7942 during FFA production.

Genetic targets for addressing FFA toxicity in FFA-producing *S. elongatus* PCC 7942 were identified from the RNA-seq analysis, targeted mutagenesis experiments, and comparative transcriptomics analysis. Differentially expressed hypothetical proteins, ROS-degrading proteins, and potential FFA transporters were targeted to generate 15 mutant strains. The overexpression of ROS-degrading proteins improved cell growth, physiology, and FFA production, presumably due to ROS degradation and the subsequent reduction in cellular damage. Gene knockout of the two porin proteins (Synpcc7942_1464 and Synpcc7942_1607) improved FFA production and cellular physiology without significantly affecting cell growth. These porin proteins may therefore participate in FFA uptake rather than export. The overexpression of 3 hypothetical proteins (Synpcc7942_1655, Synpcc7942_0122, and Synpcc7942_0900) also improved cell growth, physiology, and FFA production. In addition to these genetic targets specific to FFA production, comparative transcriptomics of cyanobacterial stress responses identified potential targets for improving cellular stress response in general. These conserved stress response targets include a high light-inducible protein (Synpcc7942_1997), a hypothetical protein (Synpcc7942_0551), heat shock protein Hsp20 (Synpcc7942_2401), NAD(P)H-quinone oxidoreductase subunit 4 (Synpcc7942_1439), RNA polymerase sigma factor SigD (Synpcc7942_0672), and Beta-Ig-H3/fasciclin (Synpcc7942_1606). By targeting these stress response genes along with the targets confirmed in the targeted mutagenesis investigation, the physiological stress associated with FFA toxicity may be alleviated in FFA-producing strains of *S. elongatus* PCC 7942. By overcoming the physiological effects of FFA production, higher FFA productivities may be attained, advancing the development of cyanobacterial based biofuels.

## Materials and methods

### Materials and strains

Chemicals used in this study were purchased from Sigma-Aldrich (spectinomycin dihydrochloride pentahydrate and 2′,7′-dichlorodihydrofluorescein diacetate (H_2_DCFDA), MP Biomedicals (ferric ammonium citrate, zinc sulfate heptahydrate, and cupric sulfate pentahydrate), Acros Organics (sodium molybdate(VI) dihydrate and cobalt(II) nitrate hexahydrate), Gold Biotechnology (isopropyl-β-D-thiogalactopyranoside: IPTG), Invitrogen/Life Technologies (SYTOX) and Fisher Scientific (all other chemicals). DNA purification kits used in strain construction include the Zyppy Plasmid Miniprep Kit and Zymoclean Gel DNA Recovery Kit (Zymo Research Corporation). Enzymes used in cloning include DNA polymerases (Taq, LongAmp Taq, and Phusion High-Fidelity), restriction enzymes, and T4 DNA ligase from New England Biolabs. Primers were synthesized by Integrated DNA Technologies. Kits for FFA quantification and RNA isolation are described below. All strains and plasmids used in this study are listed in Additional file 3: Table S5.

### Mutant strain construction

From RNA-seq analysis of 7942, SE01, and SE02, gene targets were identified as those being differentially expressed during FFA production with statistical significance among the biological replicates. Select genes were targeted for either gene knockout or gene overexpression in SE02. The knockout plasmid, pSK, was constructed from pSA, a plasmid previously used for gene expression and integration into NSII of *S. elongatus* PCC 7942 [7]. The 5′ and 3′ NSII regions of pSA were removed sequentially by *Sac*I and *Avr*II digestion and ligation to generate pSK. The knockout plasmids for each gene target were then constructed by inserting approximately 500 bp fragments of the 5′ upstream and 3′ downstream regions into the *Sac*I and *Avr*II sites of pSK (see Additional file 3: Table S6 for primers). The subsequent knockout plasmids (Additional file 3: Table S5) were transformed into SE02 using the protocol described in [7]. Gene knockout was confirmed by PCR amplification of the target gene region using the 5′ forward and 3′ reverse primers (Additional file 3: Table S6). The pSA plasmid was used for gene overexpression and integration into NSII. The target genes were amplified using the primers listed in Additional file 3: Table S5 and inserted into the *Kpn*I and *Afl*III sites of pSA. Successful cloning and integration of the target genes in the overexpression plasmid was confirmed using PCR with a forward primer homologous to the upstream promoter sequence of pSA (psacF, Additional file 3: Table S6) and the reverse primer of the target gene. The overexpression plasmids were transformed into SE02 using the protocol outlined in [7], and successful integration was confirmed by amplification of NSII (NSII5F/NSII3R) and the promoter-gene fragment (psacF/geneR) (Additional file 3: Table S6). In total, 7 knockout mutants and 10 overexpression mutants were constructed (Additional file 3: Table S5). SE02a was constructed as the control strain by inserting the empty vector, pSA, into the NSII site of SE02.

Strain stability was an issue for several of the mutant strains. Specifically, the hypothetical protein overexpression mutants SB2632#1, SB2632#2, and S1476#1 and the ROS-degrading protein overexpression mutant S1656#2 showed reduced viability after sub-culturing. With repeated rounds of sub-culturing on solid media, these mutant strains had reduced colony growth, and following 3 to 4 rounds of re-plating, these strains were no longer viable. As a result, the mutant strains SB2632#1 and S1476#1 have only 2 biological replicates, and SB2632#2 and S1656#2 mutant strains have only 1 biological replicate for the relative cell concentration and relative extracellular FFA concentration measurements.

### Cultivation conditions

All strains were grown at 30 °C with shaking at 150 rpm and approximately 60 μmol photons m^-2^ s^-1^ illumination from alternating cool white and plant fluorescent lights. Cells were transferred from BG-11/agar plates, supplemented with antibiotics (40 μg/mL spectinomycin dihydrochloride pentahydrate (Sp) and 50 μg/mL kanamycin monosulfate (Km)) as appropriate, to test tubes containing 4 mL of liquid media. After 5 to 7 days of growth, these test tube cultures were used as inoculums for both the large-scale FFA production experiments and the small-scale mutant screening experiments.

The large-scale FFA production experiments were used for obtaining samples for RNA-seq analysis. For these experiments, a second inoculum was prepared by adding 1 mL of the test tube inoculum to a 500 mL baffled Erlenmeyer flask containing 100 mL of BG-11 medium and antibiotics as appropriate. These larger inoculums were grown for approximately 4 days under the aforementioned growth conditions. These cultures were added to 1 L media bottles containing 400 mL of BG-11 media so that the optical density at 730 nm (OD_730_) was approximately 0.15. No antibiotics were added to the final experimental cultures to reduce any effects of the antibiotics on the experimental results. The large-scale cultures were grown under the previously described conditions and bubbled with air and 1% CO_2_. Additional setup information can be found in [7]. Thioesterase (*‘tesA*) expression was induced by the addition of 0.1 M isopropyl β-D-1-thiogalactopyranoside (IPTG) to a final concentration of 1 mM after approximately 4 days of growth. Samples were taken every 2 days for analysis of growth (OD_730_), photosynthetic yield (F_v_’/F_m_’), and FFA concentration. Every 4 days, samples were taken for measurement of ROS levels and cell membrane permeability. Samples for RNA-seq analysis were taken before induction (day 4) and 8 days after induction (day 12). Three biological replicates were performed for each large-scale experiment.

Small-scale experiments were conducted to screen the 7 knockout and 10 overexpression mutants. For each mutant strain, two transformed colonies were selected for screening to account for possible variation among the transformants. This was particularly important for the overexpression mutants, which may have tested positive for PCR amplification of the inserted gene but could have had a mutation effecting gene expression. For the small-scale screening experiments, 1 mL of the test tube inoculum was added to 25 mL of BG-11 medium in a 125 mL baffled Erlenmeyer flask. After 4 days of growth, cultures were induced by adding IPTG to a final concentration of 1 mM. Samples were taken every 3 days to measure growth (OD_730_), photosynthetic yield (F_v_’/F_m_’), and FFA concentration. Three biological replicates were performed for each small-scale experiment.

### Physiological measurements

Bulk spectroscopic measurements were taken to assess cell growth, photosynthetic yield, and extracellular FFA concentration. Cell growth was estimated by measuring the optical density at 730 nm (OD_730_) using a PerkinElmer Lambda Bio spectrophotometer. A linear calibration curve relating OD_730_ and gDCW was obtained previously [7]. Photosynthetic yield was measured using a Waltz MINI-PAM Photosynthetic Yield Analyzer, and the reported F_v_’/F_m_’ values are averages of 3 technical replicate measurements. To measure extracellular FFA concentration, 0.5 mL of culture was centrifuged at 5000 x g for 3 min. The supernatant was transferred to a new tube and frozen at −20 °C until analysis. FFA concentration was analyzed using the Free Fatty Acid Quantification Kit from Biovision, as described previously [7].

Single-cell analysis was used to measure the levels of ROS and cell membrane permeability in each culture. For ROS analysis, 0.5 mL of culture was mixed with H_2_DCFDA at a final concentration of 350 μM with 10 μL of dimethyl sulfoxide (DMSO) to permeabilize the cell membrane for dye uptake. The mixture was incubated under culture conditions for 1 hour to allow for dye diffusion and ROS-mediated activation. The culture was diluted and analyzed using an Accuri C6 flow cytometer. For each measurement, 20,000 cells were analyzed, and cells with an increased fluorescence emission at 533 nm were determined to be positive for ROS. To measure cell membrane permeability, SYTOX Green Nucleic Acid Stain (Invitrogen/Life Technologies) was added to 0.3 mL culture samples to a final concentration of 300 nM. After 20 min of incubation in the dark at room temperature, the dyed cultures were analyzed using flow cytometry. Approximately 20,000 cells were measured for fluorescence emission at 533 nm, and an increase in fluorescence intensity at this wavelength was indicative of positive SYTOX staining (i.e. membrane permeable cells). Both ROS detection and cell membrane permeability are reported as the percentage of the cell population with positive staining.

### Statistical data analyses

To determine the statistical significance of the relationships between measured variables, a single factor ANOVA was performed. The resulting *p*-values are reported in the text along with the R^2^ value associated with a linear regression analysis. To confirm statistically significant variation between the control strain SE02a and the targeted mutants, two-factor ANOVA analyses with replicates were performed for relative OD_730_ measurements (days 7 – 19) and relative FFA concentration (days 10 – 19). For the photosynthetic yield data, single factor ANOVA analyses were applied to the measurements from day 7, comparing the control (SE02a#1 and SE02a#3) and mutant data. Mutant strains with statistically significant (*p* < 0.05) changes relative to the control are indicated in Figures 2 and 3. The calculated *p*-values from the ANOVA analyses are listed in Table S7 (Additional file 3). The ANOVA analyses were conducted using the Analysis TookPak add-in in Microsoft Office Excel 2010.

### RNA preparation, library construction, and cDNA sequencing

RNA was extracted from 50 mL of culture using the protocol described in [15]. RNA concentration was measured in diluted RNA samples using the Quant-iT RiboGreen RNA Reagent and Kit (Invitrogen/Life Technologies), and RNA quality was assessed using an RNA 6000 Nano Kit (Agilent) and an Agilent 2100 Bioanalyzer. The Ribo-Zero rRNA Removal Kit for Gram-Negative Bacteria (Epicentre) was used to reduce the amount of ribosomal RNA (rRNA) in each sample, and the rRNA-depleted samples were purified using the RNA Clean & Concentrator – 25 Kit from Zymo Research. RNA concentration and quality were assessed again after rRNA removal. The RNA samples were stored at −80°C and sent to Los Alamos National Laboratory for library construction and cDNA sequencing. TruSeq RNA Sample Preparation Kits (Illumina) were used for library construction, and the libraries were sequenced using the Illumina HiSeq 2000. A total of 17 samples were analyzed across 3 lanes of the HiSeq 2000. This includes 3 strains (7942, SE01, SE02) at 2 different time points (day 4 and day 12) with 3 biological replicates. One replicate sample for the SE02 strain at 100 h was eliminated due to low RNA yields after rRNA removal.

### RNA-seq data analysis

A total of 524 million reads corresponding to 52.9 billion bases of sequence data were generated from the 17 RNA samples. After sequencing, the de-multiplexed data files for each sample were analyzed using FastQC v 0.10.1 [37]. The average Phred quality score per read was 36, indicating high sequence quality. The quality check also revealed several issues with the read data which were addressed by additional data processing steps. First, adapter sequences were found to be prevalent among the sample reads, despite the fact that data had been de-multiplexed using the Illumina software. Cutadapt 1.0 [38] was used to remove these persistent adapter sequences and to remove nucleotides with low quality scores (phred < 20). FastQC also identified an abundance of polyA/T sequences at the read ends. These polyA tails may be indicative of a cyanobacterial mechanism for transcript degradation [39]. Therefore, terminal polyA/T sequences were removed from the reads using Prinseq [40] to improve read alignment. Prinseq was also used to remove any reads less than 20 nucleotides. Bowtie v 0.12.7 [41] was used to align the reads to the *S. elongatus* PCC 7942 genome index. The genome index was constructed using 3 NCBI files: NC_007604.fna for the *S. elongatus* PCC 7942 chromosome, NC_007595.fna for plasmid 1, and NC_004990.fna for pUH24. If the Bowtie alignment was restricted to only uniquely mapped reads (i.e. any read aligning to more than one region of the genome is discarded), there was a significant reduction in the number of aligned reads, with more than 96% of the total reads being discarded in the most severely affected sample. As a result, the best alignment for each read was selected, and no reads were discarded due to multiple alignments to the reference genome. The SAM files constructed by Bowtie were submitted to HTSeq v. 0.5.3p3 to obtain raw counts for each predicted gene feature in the *S. elongatus* PCC 7942 genome. Raw counts for each sample are shown in Additional file 4. These feature counts were then analyzed by EdgeR for differential gene expression analysis. EdgeR includes 3 normalization methods: weighted trimmed mean of M-values (TMM), relative log expression (RLE), and upperquartile normalization [42]. All three normalization methods were applied separately, and the results for each gene feature were combined and averaged. There was good agreement of the differentially expressed genes between the three methods of normalization. EdgeR uses Fisher’s exact test to compute p-values for the calculated fold changes. Fold changes with p-values < 0.05 were considered to be statistically significant. Raw and processed data from this RNA-seq study was deposited in Gene Expression Omnibus (Accession #: GSE45762).

### Comparative transcriptomics

Differentially expressed genes associated only with comparison F were excluded from the comparative transcriptomics analysis. Comparison F (SE02 – 240 h:100 h) includes an increase in extracellular FFA concentration but no change in FFA concentration on a per cell weight basis. Furthermore, the differentially expressed genes identified in comparison F suggest a reduction in cellular stress levels, contrary to the other 5 comparisons. These reasons justify the exclusion of comparison F. The Microbial Genome Database (MBGD) for Comparative Analysis was used to identify homologs among the three cyanobacterial species: *S. elongatus* PCC 7942, *Synechococcus* sp. PCC 7002, and *Synechocystis* sp. PCC 6803. Differential gene expression data were obtained for each reference using either supplemental material or tables provided in the publications [25-33,43]. These differentially expressed genes were compared to the list of differentially expressed genes in *S. elongatus* PCC 7942 during FFA toxicity (determined in this study) using the list of homologs generated from MBGD and a Perl script generated for this comparison.

## Additional files

The following additional data files are available with the online version of this paper.

## Abbreviations

ACP: Acyl carrier protein; c-di-GMP: Cyclic-di-guanosylmonophsophate; CoA: Coenzyme A; FC: Fold change; FFA: Free fatty acid; gDCW: Grams of dry cell weight; IPTG: Isopropyl β-D-1-thiogalactopyranoside; Km: Kanamycin monosulfate; MBGD: Microbial genome database; NAD(P)H: Nicotinamide adenine dinucleotide phosphate; NSII: Neutral integration site II; PBS: Phycobilisome; PCR: Polymerase chain reaction; PEP: Phosphoenolpyruvate; PSI: Photosystem I; PSII: Photosystem II; PUFA: Polyunsaturated fatty acid; RNA: Ribonucleic acid; ROS: Reactive oxygen species; RuBisCO: Ribulose-1,5-bisphosphate carboxylase/oxygenase; SE: Standard error; SFA: Saturated fatty acid; Sp: Spectinomycin dihydrochloride pentahydrate; UFA: Unsaturated fatty acid.

## Competing interests

The author declares that she has no competing interests.

## Authors’ contributions

AMR conceived of the study, carried out the physiological studies, conducted the experiments for RNA sampling and isolation, processed and analyzed the RNA-seq data, constructed and tested the mutant strains, performed the comparative transcriptomics, interpreted the data, and drafted the manuscript.

## Supplementary Material

Additional file 1: Table S1 Listing the genes up-regulated during FFA production. **Table S2**, listing the genes down-regulated during FFA production. Click here for file

Additional file 3: Table S5. Listing the strains and plasmids used in this study. **Table S6**, listing the primers used in this study. **Table S7**, listing the *p*-values for the ANOVA analyses. Click here for file

Additional file 2: Table S3 Listing the up-regulated genes from the comparative transcriptomics analysis. **Table S4**, listing the down-regulated genes from the comparative transcriptomics analysis. Click here for file

Additional file 4: **Contains the raw counts for each biological sample analyzed using RNA-seq**. Click here for file
